# How Older Adults and Their Families Perceive Family Talk about Aging-Related EOL Issues: A Dialectical Analysis

**DOI:** 10.3390/bs7020021

**Published:** 2017-04-17

**Authors:** Nichole Egbert, Jeffrey T. Child, Mei-Chen Lin, Carol Savery, Tammy Bosley

**Affiliations:** School of Communication Studies, Kent State University, Kent, OH 44242-001, USA; jchild@kent.edu (J.T.C.); mlin@kent.edu (M-C.L.); csavery@kent.edu (C.S.); tbosley5@kent.edu (T.B.)

**Keywords:** end-of-life (EOL), relational dialectics theory (RDT), older adults (OA), aging, families

## Abstract

For older adults, approaching end-of-life (EOL) brings unique transitions related to family relationships. Unfortunately, most families greatly underestimate the need to discuss these difficult issues. For example, parents approaching EOL issues often struggle with receiving assistance from others, avoiding family conflict, and maintaining their sense of personhood. In addition, discussions of EOL issues force family members to face their parents’ mortality, which can be particularly difficult for adult children to process emotionally. This study explored aging issues identified by aging parents and their families as they traverse these impending EOL changes. Ten focus groups of seniors (*n* = 65) were conducted. Focus groups were organized according to race (African-American/European-American), gender, and whether the older adult was living independently or in an assisted care facility. When asked open-ended questions about discussing aging and EOL issues with family members, participants revealed tensions that led us to consider Relational Dialectics Theory as a framework for analysis. The predominant tension highlighted in this report was certainty versus uncertainty, with the two sub-themes of sustained life versus sustained personhood and confronting versus avoiding EOL issues. For these data, there were more similarities than differences as a result of gender, race, or living situation than one might expect, although culture and financial status were found to be influential in the avoidance of EOL discussions. The results of this study help to provide additional insight into relational dialectics related to aging, EOL, and the importance of communication in facilitating family coping.

## 1. Introduction 

In 2014, the number of Americans 65 years and older was 45.2 million. By 2060, this group will number 98.2 million, nearly one in four U.S. residents [1]. Based on these numbers, one would think that aging and end-of-life (EOL) issues would inhabit a prominent place in American discourse. However, older individuals and their families still greatly underestimate the need to discuss end-of-life (EOL) issues, even when family members approach old age. Fewer than 50% of adult children discuss EOL issues with their aging parents [2,3]. There are various explanations for why families avoid these discussions. Hummert and Morgan [4] suggest that discussions of aging-related issues may force family members to face unavoidable issues related to mortality, which can be particularly difficult for adult children to process emotionally. Old age also creates uncertainty for parents as they struggle between receiving assistance from others, maintaining personal independence, and providing continuous nurturance and support to other family members [5]. To investigate this issue further, this study explores the tensions felt by older adults and their families and how the tensions constrain and shape conversations about EOL issues. 

Aging-related EOL negotiations and decisions are clearly communicative in nature and thus are best understood through the lens of communication theory. Relational Dialectics Theory (RDT) is well-suited for investigating the interplay of opposing forces such as those experienced by older adults and their families regarding the discussion of EOL issues [6]. “Dialectical tensions are the conflicts felt between two or more opposing forces, frames, or themes” [6] (p. 548). Although RDT conceptualizes dialectical tensions as opposites, other factors can also play important roles. For example, the interdependent nature of the opposing forces is equally critical. In other words, there cannot be tension or interplay without interdependence. In essence, the elements are unified at the same time they are opposites. 

Thus, RDT helps us to understand how we construct meaning for our relational experiences through the interplay of competing discourses [7]. Relationships, themselves, are created through conversation [8]. Furthermore, Baxter and Norwood [7] emphasize that conversation includes an utterance chain, which means that all dialogue is connected to the past and the future. The utterance chain demonstrates that RDT is not linear. The conversational aspect highlights both the history and the ongoing nature of the relationship. Additionally, we see instances of power and struggle within discourse. However, Baxter and Norwood [7] do not see these two components as negative. Power and struggle are important because they lead relationships into what Baxter and Norwood describe as turning points. 

A turning point is some sort of change in the relationship brought on by a transformative event, such as an illness or relocation due to aging [6,9]. Turning points, arguably, provide a better explanation than stage models in terms of understanding how relationships progress over time [10]. Turning points can be positive or negative, or a combination of both. For example, a tragedy might prompt relational partners to be grateful for their relationship. Interestingly, relational partners do not necessarily identify the same turning points. 

RDT is an elegantly simple theory [7]. The core components of the theory (e.g., dialectical tensions and turning points) provide the appropriate foundation to investigate what happens to relationships as we age and face major turning points in life, such as confronting EOL decisions. To that end, we offer the following research questions:

RQ_1_: What are the inherent tensions embedded in the discourse of older adults with their families about end-of-life (EOL) issues?

Although it is informative to investigate this discourse broadly, as researchers, we also understand the heterogeneity of human experience, especially as it exists in the experiences of different cultural groups. To this end, Morycz [11] argues that “each ethnic and minority group retains its own individual meaning of family structure and family caregiving” (p. 68). One’s cultural understanding of old age or EOL affects how family members approach these topics in conversation. For example, although younger family members from Eastern cultures such as China and Japan report more respect for their elders than younger adults from Western cultures, they also find intergenerational communication more problematic [12]. Latinos living in the U.S. are incredibly family-centric, but avoid EOL discussions to a much greater extent than white, non-Latinos in the U.S. [13]. 

Along the same lines, cultural understandings of both “family” and “gender” are always changing, however, there is still recent evidence that family caregivers tend to be female and that women do more domestic labor and are more likely to seek medical care than men [14]. Thus, in keeping with previous research that has found differences between ethnic and gender groups with regard to aging issues [15,16], we explore:

RQ_2_: How do these inherent tensions differ based on the gender, ethnicity (African American/European-American), and living situation (living independently/living in a residential care facility) of older adults?

## 2. Materials and Methods

### 2.1. Participants 

Participants were purposely selected and placed into one of eight distinct focus groups using a theoretical sampling technique. Because the two original focus groups of the independent-living African American (A-A) participants were much smaller than the other groups, we added two more groups—one additional group for A-A independent-living women, and one additional group for A-A independent-living men. In addition, the experiences of individuals who live on their own were expected to differ markedly from those of individuals who require daily assistance in their activities. Thus, four focus groups were planned with independent-living seniors, and four with assisted-living seniors living in a long-term care facility. Separate focus groups were arranged according to race, and male and female focus groups were also held independently. In the end, the eight focus groups were as follows: independent-living A-A men (2 groups), independent-living European-American (E-A) men, independent-living A-A women (2 groups), independent-living E-A women, assisted-living A-A men, assisted-living E-A men, assisted-living A-A women, and assisted-living E-A women. These eight groups, along with two doubled groups, yielded a total of ten focus groups. 

To recruit focus group participants, we contacted area senior centers, churches, nursing homes, and assisted living homes. A member of each participating organization then advertised the study to members. In total, participants were recruited from one senior center (*n* = 37), one church group (*n* = 11), and two nursing home/long-term care facilities (*n* = 17). In all, 65 seniors participated in the ten focus groups, averaging approximately six to seven individuals per group. The study included 33 female participants (50.8% of the sample) and 32 male participants (49.2%). The average focus group participant age was 75 years old (*M* = 74.95; *SD* = 9.98). Regarding marital status, the majority of participants was still married (34 participants, 52.3%), with 17 seniors widowed (26.2%), nine seniors single (13.8%), and five seniors divorced (7.7%). The average senior had three children (*M* = 3.28; *SD* = 2.27). Educational attainment was varied, with 14 seniors (22%) having some high school, 20 (31.7%) with a high school diploma, 13 (22.2%) having some college, and 15 (23.8%) with a graduate degree or some post-graduate education. Focus group members received a $25 gift card for their participation in the study.

### 2.2. Procedures 

To increase comfort and self-disclosure in focus groups, the researchers used gender and ethnicity to determine focus group membership [17]. In each case, the focus group moderator was over 50 years old and matched to the focus group participants according to ethnicity, gender, or both ethnicity and gender. The focus group moderators were experienced interviewers who were well-versed in the goals and dynamics of qualitative focus group interviews. 

### 2.3. Data Analysis 

The researchers used a semi-structured interview protocol to guide the focus group discussion. Open-ended questions sparked conversation among participants and enabled them to build on one another’s responses, thus yielding more natural data. Probes were used when necessary to clarify participants’ responses. The focus groups each lasted approximately 60–90 min, and were audiotaped and transcribed verbatim for further analysis. In the end, the transcripts comprised 159 single-spaced pages of text. 

The researchers used open, axial, and selective coding to analyze the data [18]. During the first phase of open coding, the researchers individually read through the transcripts and identified emergent categories of dialectics. The researchers then compared their codes by applying them to the first focus group transcript and developing a codebook, which was subsequently used to analyze and interpret the remaining transcripts. After discussing the fit of these codes and making revisions where necessary, the codebook was finalized.

Once the codebook was finalized, the researchers coded the transcripts using axial coding, a strategy in which a set of defined codes are applied to the data. The unit of analysis was a turn of talk, and more than one code could be applied to each turn of talk. However, no matter how many times a participant discussed a particular code in a turn of talk, each code was only counted once per unit of analysis. Two researchers independently coded each transcript. Any disagreements on the fit of codes were discussed among all researchers until consensus was reached. 

## 3. Results 

The focus of this current study was on EOL issues (specifically wills and distribution of property, living wills, funerals, etc.). Older adults discussed how they communicated (or did not communicate) with family members about these topics. The prominent dialectic that colored these focus group narratives was that of certainty versus uncertainty. This theme depicts the opposing desires of knowing exactly what is happening or going to happen versus not worrying about the unknown. (1) *Sustained life* versus *sustained personhood* and (2) *avoiding aging and end-of-life issues* versus *confronting them* were prominent sub-themes for this dialectic. Previous research suggests that individuals differ in their demands for certainty and uncertainty, and that managing uncertainty is vital for relational well-being [19]. 

Often, participants’ communication with family members was viewed as an important tool for dealing with the uncertainty of EOL. An independent-living, A-A woman shared, “I saw how quickly changes come about. How unpredictable life is, you know, and that everybody needs to be prepared in the family, you know, and that, and I constantly talk to my family.” Sometimes older adults’ uncertainty can make it difficult for family members to provide support. For example, an independent-living, E-A female shared how uncomfortable this uncertainty is for both her and her daughter, “Our daughter wants to know if she’s doing what Mom would be happiest with and Mom doesn’t know what she’d be happiest with.”

On the other hand, seniors without strong family bonds may turn to others to help cope with uncertainty about EOL. For instance, there were voices that expressed gratitude for the certainty of life in a care facility, as described by one assisted-living A-A woman, “We don’t know how we [are] gonna be treated at home. But you know when you come in here and be here a couple of weeks, somethin’ like, you know how you gonna be treated.” Thus, the dialectic of certainty versus uncertainty was often expressed as a relative concept, with participants weighing their current set of circumstances against another perceived situation, either one that they witnessed in the lives of other people or one that they, themselves, had previously experienced.

### 3.1. Sustained Life Versus Sustained Personhood 

This sub-category was described as the struggle for older adults to choose between factors that may sustain their physical lives or their overall personhood by either allowing or not allowing extensive medical interventions at the EOL stage. For example, one independent-living E-A male was cognizant of making decisions about quality of life, “And uh, I made up my mind I went to see my wife’s uncle in Medina, and he was on the iron lung that was keeping him alive… Don’t you ever do that to me. I said that’s not living…” The importance of communicating quality of life desires to the family was reiterated by an independent-living A-A female, “I’ve talked with my children and siblings… I wouldn’t want to be on life support unless it was something that would make a positive difference.” The word “unless” in the second excerpt clearly suggests the dialectic tension as her responses switched back and forth between sustaining life and sustaining personhood. 

The sustained life versus sustained personhood tensions were also linked to the uncertainty/certainty of respecting religious beliefs. An independent-living A-A female discussed the sanctity of her beliefs even though her husband did not share them, 

“My children, three of my children are Jehovah’s Witnesses and three of my immediate family members are Witnesses, so you know, I know they will be there… My husband is very much aware of my wishes that I want, only be on the machine if the neurologist says, well there’s some brain waves, she may come through. But longer than a week, I think that a punishment. Don’t keep me on that machine… I know they will all be there and do what I asked them to do, simply because they are of the same faith.”

In this case, it is not only the woman’s personhood that was at stake, but her ability to do what she saw as consonant with God’s will. 

### 3.2. Avoiding Aging and EOL Issues Versus Confronting Them

This tension was described as evading thoughts or conversations about aging and death versus dealing with them directly within the family unit. The E-A focus group members (regardless of living situation) typically spoke of ways in which their EOL plans were in order. One independent-living E-A woman shared, “I talk with my daughter, my son too, but my daughter especially, with anything. They got all my papers, double my insurance. They got everything in the safety deposit box.” On the other hand, an independent-living A-A female discussed her mixed feelings in confronting EOL issues, “I’m with the group that, uh, contemplates it and has not just gotten around to it yet. Looked at it, talked about it, read the entire booklet, and looked at it but just didn’t act on it.” Avoidance of EOL issues was shared by an independent-living A-A male who discussed preparing a living will, “I haven’t done any of this. I mean, I’ve done a lot of talkin’ but in terms of puttin’ things down on paper, I think I’m too young to be. But it looks like I better start.” 

Other older adults shared stories of reluctance to communicate about EOL issues, either from the family or within themselves. An independent-living A-A female recounted, “Basically, they don’t want to hear it because it is like, I’m planning my demise or something, you know? And I’m saying, really I’m just trying to prepare you guys in case I do leave this world early.” One of the A-A women in the assisted-living home spoke of not wanting to communicate at all about EOL issues, “I didn’t even talk about it. I haven’t talked about it. Whatever they want to do after I’m gone, do it… If you gonna burn me, burn me, if you gonna put me in the ground, put me in the ground. I don’t know anything about it anyway.” 

When comparing the transcripts of the A-A focus groups with the E-A focus groups, there were more similarities than differences. Recent research shows that both E-A and A-A elders prefer to remain at home until the demands of their care exceed personal and family resources [20]. That being said, the independent-living African Americans in our focus groups spoke vehemently against the idea of nursing homes. One independent-living A-A woman said she was open to the idea but her family would not hear of it, “To me it would be sound to put me in one place and then you come and visit and see to me. Oh no, oh no, no you’re not goin’ there.” Another woman from the same group concurred, “There’s no way. [My daughter] would die first before she put me in a nursing home.” This aspect of African-American culture was brought to the forefront in the independent-living A-A male focus groups, regarding avoidance of EOL planning. One independent-living A-A man explained that, “Basically and this is what happens to a lot of us in the Black community and the Black culture, is that we never prepare ourselves for the day we go into the ground.” 

## 4. Discussion

The findings of this study easily merge with the larger corpus of relational dialectic research dealing with families. Talk about relationships is a vital part of the coping process for families during relationship turning points [21]. Toller [22] provided some more specific advice for families in transition, such as teaching others how to communicate with you, taking initiative in conversations, communicating needs clearly, and being open and willing to talk about important issues even when you are not comfortable. Conflict is another important aspect of relationship change, so when it arises between family members, it is important to recognize opposing needs and manage them through discourse, conflict resolution, or mediation [23]. In the case of these families of older adults, both parties frequently reported avoiding conversations about EOL because they did not want to think about the death of their loved one. Many people fear that bringing up taboo topics would damage relationships or bring about some other negative outcome [22,24]. Alternatively, Zhang and Siminoff [25] suggest that silence serves to protect the other person (or oneself) from psychological stress and emphasizes positive thinking. Unfortunately, avoiding discussions of EOL only delays the confusion and conflict until another day, which may be precipitated by a destabilizing life event such as a major illness or accident. Although one may never be fully prepared for the changing family dynamics of older adulthood, direct communication about relationships and EOL can allow family members to negotiate some of the more pressing relational tensions before a crisis occurs. 

Relational Dialectics Theory provided a helpful lens for considerations of the multiple competing goals that exist in all family relationships, but especially in situations involving older adults. Other helpful theoretical perspectives have also been used to lend insight to EOL family issues, such as Politeness Theory [26], Communication Privacy Management Theory [27], and the Theory of Motivated Information Management [28]. These studies and others that focus on family communication at EOL all grapple with when and how to initiate difficult conversations related to aging and EOL. Although these various theories differ somewhat in their strategies, they share an emphasis on the importance of collaboration and mutual respect in these family discussions.

Any distinctions based on gender, living status or race/culture in this study are only exploratory, as the study included only a small sample of individuals from one geographic region in northeast Ohio. African-American reticence may have roots in cultural distrust of local residential care agencies. However, research shows that A-A residents are more likely to report experiences and fears of discrimination occurring in long-term care facilities [20]. Culture, poverty, as well as historical discrimination and mistreatment on the basis of race may partially explain why only 27% of A-A nursing home residents have a living will as compared with 63% of E-A older adults residing in nursing homes [29]. Thus, culture and financial status may still play an important role in how older adults communicate about EOL issues with family members. Based on our findings, we did not determine that gender played a significant role in perceptions of family conversations related to EOL. 

## 5. Limitations 

Based on these transcripts and the subsequent analysis, conversations about EOL need to be understood in the broader context of family communication. The conclusions derived from these ten focus groups are not intended to be generalizable, but rather illustrative of the complex and multi-layered tensions experienced by older adults and their families. For example, although we sought to acknowledge the roles of ethnicity and culture in aging by including both African American and European American seniors in our study, other geographical, cultural, and/or situated groups of seniors may experience tensions not named here. In addition, the arrangement of focus groups that isolated participants based on gender, race/culture, and living status may have de-emphasized the similarities that are shared amongst these groups. However, family research on families of color in later life is scant at best and in need of further exploration [30].
